# Asymmetric Cell Division in Polyploid Giant Cancer Cells and Low Eukaryotic Cells

**DOI:** 10.1155/2014/432652

**Published:** 2014-06-22

**Authors:** Dan Zhang, Yijia Wang, Shiwu Zhang

**Affiliations:** ^1^Department of Pathology, Tianjin Union Medicine Center (Nankai University Affiliated Hospital), Tianjin 300121, China; ^2^Clinical and Translation Medicine Lab, Tianjin Union Medicine Center, Tianjin 300121, China

## Abstract

Asymmetric cell division is critical for generating cell diversity in low eukaryotic organisms. We previously have reported that polyploid giant cancer cells (PGCCs) induced by cobalt chloride demonstrate the ability to use an evolutionarily conserved process for renewal and fast reproduction, which is normally confined to simpler organisms. The budding yeast, *Saccharomyces cerevisiae*, which reproduces by asymmetric cell division, has long been a model for asymmetric cell division studies. PGCCs produce daughter cells asymmetrically in a manner similar to yeast, in that both use budding for cell polarization and cytokinesis. Here, we review the results of recent studies and discuss the similarities in the budding process between yeast and PGCCs.

## 1. Introduction 

Asymmetric cell division is essential for generating cell diversity during development in low-level eukaryotes, including yeast. The budding yeast,* Saccharomyces cerevisiae*, has served as an excellent model for studying this process [1]. In animals, stem cells have the ability to undergo an asymmetrical, self-renewing cell division, resulting in one stem cell and one more differentiated progenitor cell [2].

Polyploid giant cancer cells (PGCCs) are key contributors to the cellular heterogeneity observed in human solid tumors. We have successfully purified and cultured PGCCs from 22 kinds of cancer and immortalized cell lines. PGCCs meet the definitions of cancer stem cells and play a fundamental role in regulating heterogeneity, stemness, and chemoresistance among human solid tumor cells. Single PGCCs formed cancer spheroids* in vitro* and generated tumors in immunodeficient mice, demonstrating that PGCCs have cancer stem cell-like properties. The PGCCs were slow-cycling in nature and stained positively for both normal stem cell and cancer stem cell markers. They were prone to differentiate into other tissue types, including adipose, cartilage, and bone, and were found to generate regular cancer cells through the budding, splitting or burst-like mechanisms common in the replication of low-level eukaryotes, including yeast [3–5]. In this review, we review the possible molecular mechanism of asymmetric cell division in lower eukaryotic cells and PGCCs.

## 2. Polyploid Giant Cells

Polyploidy refers to a karyotypic state where the chromosome number is a multiple of the chromosome number of the gamete [6]. It gives rise to chromosomal instability, as seen in a high rate of chromosomal division errors. Polyploidy is an important cause of human reproductive diseases, such as infertility, spontaneous abortions, and congenital birth defects, with data showing that about 20% of spontaneous abortions are caused by polyploidy [7]. Polyploidy are considered as being on the verge of mitotic catastrophe and subsequent apoptosis [8].

## 3. Polyploid Giant Cells and Cancer

As long as a century ago, it was found that some tumor cells often have extra chromosomes. Normal human cells contain 46 chromosomes but tumors cells contain abnormal numbers (usually between 60 and 90), with cell-to-cell variability. Structural abnormalities such as inversions, deletions, duplications, and translocations are commonly observed in these chromosomes but are rare in normal cells, and PGCCs are key contributors to the heterogeneity of human solid tumors. By and large, however, PGCCs have not attracted the attention they deserve from the cancer research community because of their poorly understood biological role in cancer. Studies have reported a close relationship between the proportion of PGCCs in tumors and tumor deterioration, risk of metastasis [9], treatment effectiveness, and recurrence rate [10, 11]. The relationship between polyploidy and cancer has long been known, but it is not clear if polyploidy is a contributing factor to tumorigenesis or only a consequence of malignant transformation [12, 13].

Clinical evidence is accumulating in support of the idea that polyploidy positively contributes to tumorigenesis. First, polyploidy occurs before transformation.* In vivo*, polyploid cells exist in many precancerous tissues, such as the cervix [14], head and neck [15], colon [16], esophagus, and bone marrow [17]. Polyploid cells are also observed in breast [18] and skin tissues of experimental animals [19]. Second, polyploidy disturbs the overall transcription level, upregulating genes promoting cell growth and downregulating cytostatic genes. Tumorigenesis and transformation caused by polyploidy need many rearrangements to build the complex karyotype of tumor cells. Expression errors in tumor cell genes contribute to unrestricted growth, which is similar to the upward trend in the tumorigenesis rate that occurs with increasing age [20]. Polyploidy, rather than a cellular genetic phenomenon, is necessary for tumorigenesis [21, 22]. Tetraploidy might enhance tumorigenesis by the buffering effects of additional normal chromosomes. Extra chromosome sets might mask the effects of deleterious mutations if these mutations are recessive or partially recessive, thereby allowing cells with DNA damage to survive longer until a crucial growth-enhancing or transforming mutation occurs. This effect of increased ploidy has been best studied in yeast evolution experiments [23]. Diploid budding yeast mutator strains defective in mismatch repair have a significant advantage over haploid mutators in long-term evolution experiments [23]. Besides increasing tumorigenesis, polyploidy is also a contributing factor to or an incidental product of cell malignant transformation [24].

## 4. PGCCs and Cancer Stem Cells

In cancer, multiple stresses, including antimitotic chemotherapy drugs, radiotherapy, hypoxia, or poor microenvironment, can increase the formation of PGCCs. PGCCs with slow-cycling nature stain positively for normal and cancer stem cell markers. These cells are prone to differentiation into other tissues and cell types, including adipose, cartilage, erythrocytes, fibroblasts, and bone [3–5, 25]. Single PGCCs form cancer spheroids* in vitro* and generate tumors in immunodeficient mice, whereas large numbers (hundreds) of regular cancer cells do not, demonstrating that PGCCs have cancer stem cell-like properties. Proteomic analysis of PGCCs reveals a distinct signature, involving proteins related to hypoxia, invasion, chromatin-remodeling, and cell cycle regulation [3]. Thus, PGCCs may exhibit an evolutionarily conserved mechanism that cancer cells use to achieve malignant growth through increased cell size and highly efficient replication. PGCCs play a fundamental role in regulating heterogeneity, stemness, and chemoresistance in solid human cancers [5].

Cancer stem cells are a small subset of cancer cells that are capable of generating entire tumors [26, 27]. To date, stem cell-like populations have been characterized using cell-surface protein markers in tumors [28]. The nature of such so-called stem cells remains disputed, however [29, 30]. The American Association for Cancer Research consensus conference workshop broadly defined a cancer stem cell as “a cell within a tumor that possesses the capacity to self-renew and to cause the heterogeneous lineages of cancer cells that comprise the tumor [2].” Single cells in mice that generate tumors represent the gold standard for cancer stem cells. Cancer stem cells also have slow cycles, exhibit asymmetric division, and have the unique potential to divide asymmetrically to generate daughter cells with different fates, one of which remains a stem cell and the other turns into a cell committed to tumor formation [31]. By dividing asymmetrically, cancer stem cells maintain the stem cell pool and simultaneously generate committed cells that form tumor mass [32]. Many secrets of the cell cycle have been resolved by studying the asymmetric division of cancer stem cells in which cytoplasmic structures like the midbody are often inherited by only one of the two daughters.

## 5. Asymmetric Cell Division of PGCCs in Cancer 

In multicellular eukaryotes, mitosis is the recognized process for somatic cell division, ensuring the accurate separation of duplicated genetic material to progeny cells. As a result, eukaryotes have well-regulated and orderly growth, with a low mutation frequency. In contrast, prokaryotes and unicellular eukaryotes divide by amitotic processes, including binary fission and budding. Although mitosis predominates in complex eukaryotes, it is well documented that depending on the organism or cell type, variations can occur in the mitotic cell cycle to replicate cells and meet growth and developmental needs [33, 34]. Among these variations is the endocycle (or endoreduplication), a variation of the normal mitotic cell cycle involving multiple rounds of DNA replication. This process is commonly employed in certain forms of growth in plants, insects [33, 35–37], and trophoblasts and in the generation of platelets from megakaryocytes in mammals [34, 37]. David von Hansemann proposed that abnormal mitosis occurs in polyploid tumor cells. He found dividing cells with chromosomes of abnormal configuration and size by observing various tumor tissue sections [38]. Two chromosome configurations were mentioned by Hansemann as follows: late bridges and multipole splitting. Both of these can result in abnormal chromosome numbers and the phenomenon of heterozygosity loss caused by missing unstable chromosomes [39]. After Hansemann, Theodor Boveri, a German cell biologist and zoologist, found multipolar spindles and aneuploid daughter cells. In 1902 and 1914, he propounded the hypothesis that the generation of polyploidy leads to tumorigenesis and malignancy and is unrelated to the origins of abnormal chromosome constitution [40].

We previously reported that PGCCs can be induced and purified by CoCl_2_. These cells were found to be in a dynamic equilibrium with regular cancer cells and could be formed through endoreduplication or cell fusion [5]. They reverted to regular cancer cells via asymmetric cell divisions, including the splitting, budding, or burst-like mechanisms commonly used in the replication of low-level eukaryotes, plants, and viruses [5]. In fact, these giant cells revert to regular-sized cancer cells through a process of reductive division named depolyploidization [37, 41]. Asymmetric cell division of giant cancer cells by meiosis-like depolyploidization had been previously proposed to explain the unexpected life cycle of these cells [35, 36]. This mechanism by which PGCCs generate daughter cells has also been reported in the normal growth of skeletal muscle, osteoclasts, viral infection, and even tissue culture.

Asymmetric cell division is a fundamental process, whereby the asymmetric inheritance of cellular components defines distinct fates for each daughter cell. In a typical outcome, the stem or progenitor cell generates a copy of itself and a second daughter cell programmed to differentiate into a nonstem cell type [42]. Thus, by balancing self-renewal with differentiation, asymmetric divisions maintain the stem and progenitor cell pool while allowing the generation of diverse functional cells. Asymmetric division is a key mechanism ensuring tissue homeostasis. In normal stem and progenitor cells, asymmetric cell division balances proliferation and self-renewal with cell-cycle exit and differentiation. Disruption of asymmetric cell division leads to aberrant self-renewal and impairs differentiation. In normal, nontumor stem cells, a number of genes like* Bmi-1*,* Wnt*, and* Notch* have been described, which are responsible for self-renewal capacity. These genes have also been discovered in cancer stem cells, and their aberrant expression has been demonstrated to be essential for the formation of tumor cell mass [43]. Asymmetric cell division plays an important role in producing cell diversity during normal tissue development [44]. In principle, there are two mechanisms involved in asymmetric cell divisions. One is extrinsic asymmetric cell division, in which the daughter cells are initially equivalent, but a difference is induced by surrounding cells—the microenvironment—and the precursor cell; the second is intrinsic asymmetric cell division, in which the daughter cells are inherently different at the time of division of the mother cell [45]. Intrinsic asymmetric cell division does not depend on interactions between the daughter cells and the surrounding cells, relying instead on the different locations of proteins, RNA transcripts, and macromolecules in the daughter cells that cause each cell to assume a separate fate from that of its sibling.

## 6. Cell Cycle-Related Proteins and Asymmetric Division 

**Figure 1:**
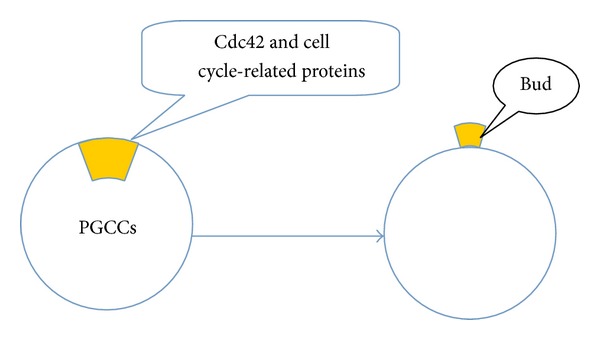
Asymmetric cell division in PGCCs. Cdc42 and cell cycle-related proteins involved in the process of PGCCs generating daughter cells.

Cyclins are regulatory subunits of cyclin-dependent kinases. The abnormal expression of cyclin-related proteins is important in the formation of stem cells. De Luca et al. confirmed that cyclin D3, a member of the mitogen-activated D-type cyclin family, is critically required for proper developmental progression in skeletal muscle stem cells [46]. Cyclin A, the first cyclin to be cloned, is thought to be a component of the cell-cycle engine whose function is essential for cell-cycle progression in hematopoietic and embryonic stem cells [47]. Our previous results also showed that cell cycle-related proteins are involved in PGCC formation [5]. These proteins, including FOXM1, Chk1, Chk2, cyclin A2, cyclin E, cyclin B1, and CDK6, play important roles in regulating the asymmetric division of PGCCs generating daughter cells (Figure 1). Expression levels of cyclin E and cyclin D1 were markedly elevated in purified PGCCs compared with that in diploid cancer cells. In particular, cyclin B1 was expressed only in the cytoplasm of PGCCs from human high-grade serous carcinomas and metastatic ovarian cancers, but had scant nuclear expression in low-grade serous ovarian cancers and no expression in benign ovarian serous cystadenomas, demonstrating that PGCC formation is regulated by recompartmentalization of cell cycle regulatory proteins normally involved in the regulation of asymmetric division [5].

## 7. Asymmetric Cell Division in Yeast

Yeast has both asexual and sexual modes of reproduction. Budding is one of the asexual modes that has long been a model in studies of cellular asymmetry aiming to discover the general principles of eukaryotic cell polarization and cytokinesis, both of which occur in yeast. Budding is a special kind of cell polarization adopted by yeast in order to undergo asymmetric cell division [48].

**Figure 2:**
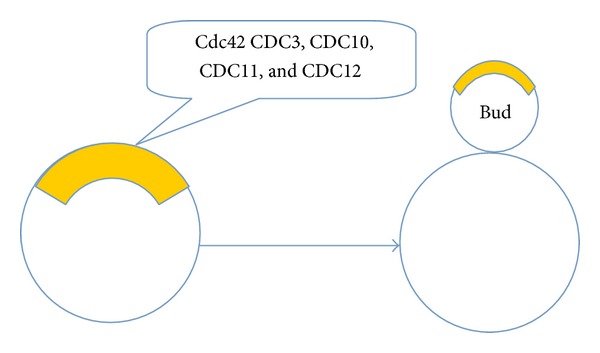
Asymmetric cell division in yeast. Cdc42 and other molecules including CDC3, CDC10, CDC11, and CDC12 locate in the special sites that will generate bud growth.

Cell polarity has been observed in almost all cells, with different cell types employing it in different ways. The mother cell divides asymmetrically by producing buds that can grow into daughter cells when they detach after cytokinesis. Polarity relies on the active determinants that localize to the plasma membrane and are associated with cell shape, cell adhesion and migration, cell division, and the uptake and release of molecules. In the polarized cell system, yeast exhibits asymmetry both in signaling molecule distribution and cytoskeleton organization. Before budding, the yeast cytoskeleton and membrane trafficking machinery become polarized to deliver cargo to the buds and then promote their growth into daughter cells [49]. The master regulator of cell polarity in budding yeast is the small GTPase, Cdc42 (cell division control protein 42). This plays a central role in cell polarization from yeasts to humans [50, 51]. It was first discovered in the yeast* Saccharomyces cerevisiae* [52]. There are six types of Rho-type GTPases in yeast, namely Rho1–Rho5 and Cdc42. They locate to the cell membrane, where they establish and maintain cell polarity. Cdc42 is critical for budding and polarization growth [52, 53]; this was initially recognized in a temperature-sensitive mutant for polarized actin organization and cell growth [54, 55]. Homologs from other species share 80–85% identity in amino acid sequence and functionally complement yeast* cdc42* mutants [55]. Cdc42 is a master regulator of cell polarization. The protein contains a C-terminal CAAX-linked geranylgeranyl membrane anchor and is uniformly distributed around the plasma membrane in symmetric interphase cells, as well as being present in the cytoplasm [48]. Loss of Cdc42 activity causes cells to grow without budding. Isotropic and polarized distribution of Cdc42 in yeast is required for polarized organization of the cytoskeleton and membrane trafficking system. In recent years, it has been shown that the cytoskeleton and membrane trafficking system are in turn able to impact Cdc42 distribution [56–58]. Actin redistribution in yeast is a dynamic process that is also regulated by Cdc42 [59]. Polarized morphogenesis is a critical process for determining the specialized functions and physiologies of cells and organs. During these processes, Cdc42 localizes to a small cortical domain that can become the bud or Shamoo site (Figure 2) [60, 61]. Here, it can impact morphological development by controlling oriented actin cables that direct both transportation of membrane vesicles and organelles and the assembly of septin. Members of the septin family, such as CDC3, CDC10, CDC11, and CDC12, are distributed to the special sites that will generate bud growth and are involved in the selection of budding sites [51, 62].

Cytokinesis is another component of the process of asymmetric cell division and plays an important role in increasing cell numbers and cell diversity during development [63–65]. It is carried out by contraction of the contractile actomyosin ring (AMR), followed by centripetal growth of the primary septum (PS) [66]. At the end of PS formation, two secondary septa (SS) are synthesized on either side of the PS. The PS and a portion of SS are then degraded by endochitinase and glucanases from the daughter side, resulting in cell separation [66]. The AMR generates contractile power that is thought to be involved in guiding membrane deposition and formation of the primary septum [66, 67]. The functions of the AMR and the PS are interdependent [68], in that the disruption of the AMR causes severely misoriented PS formation [67], and disruption of PS formation results in abnormal AMR contraction [68]. In* S. cerevisiae*, there are six families of proteins involved in AMR assembly: septins, Myo1, Mlc1, Iqg1, Bni1, and actin. Septins are the first to arrive at the division site, and their presence ensures that the other cytokinesis proteins also localize there. The members of the septin family are distributed to special sites that will generate bud growth. Septins form polymers [52, 68]. In temperature-sensitive mutants of any member of the septin family, polymerization does not occur, cytokinesis is blocked, and mitosis may proceed with the formation of multinucleated cells, a process that is similar to that forming some PGCCs. Septin1 is one of the important regulators that in mammals localizes to the mitotic contractile ring and participates in cytokinesis [52].

## 8. Asymmetric Cell Division in* Drosophila melanogaster* and* Caenorhabditis elegans*

**Figure 3:**
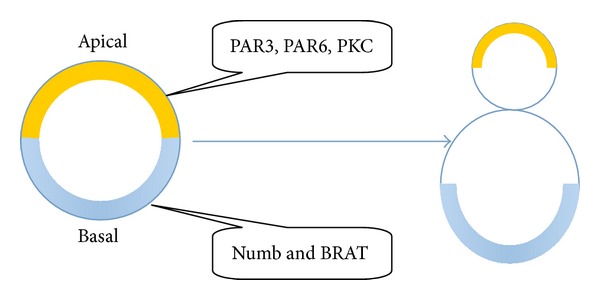
In* D. melanogaster* cells, Numb and BRAT transiently accumulate at the basal plasma membrane in the late prometaphase. Before mitosis, PAR3, PAR6, and PKC accumulate at the apical cell cortex and regulate the process of asymmetric cell division.

In addition to work on asymmetric cell division in yeast, there have been other studies, mostly in invertebrates (*D. melanogaster* and* C. elegans*). In 1994, an asymmetrically segregating cell-fate determinant was found in* D. melanogaster* and named Numb [69]. This endocytic protein (which inhibits Notch-Delta signaling) was found localized at cell margins during mitosis and segregated to only one of the two daughter cells [70]. This work also implied that high levels of Numb can cause one of the daughter cells to divide asymmetrically. Most studies on asymmetric cell division in* D. melanogaster* were done with neuroblasts [30, 71–73]. Numb and the translation inhibitor brain tumor (BRAT) transiently accumulate at the basal plasma membrane in the late prometaphase [70, 74, 75]. Before mitosis, proteins of another type, including the PDZ domain-containing proteins PAR3 and PAR6 (PAR3 and PAR6 are mutants of which are partitioning defective) and the atypical protein kinase, PKC, are required to accumulate at the apical cell cortex (Figure 3). These are involved in the asymmetric localization of basal determinants, for which asymmetric phosphorylation is the key mechanism behind the asymmetric segregation of cell fate determinants [76]. Establishing and maintaining apicobasal polarity requires apical localization of PAR proteins. It was shown that PAR3, PAR6, PKC, and their homologs play a central role in almost all known cell polarity events, including epithelial polarity, axon outgrowth, synapse formation, and specification of the anteroposterior body axis [77, 78].

**Figure 4:**
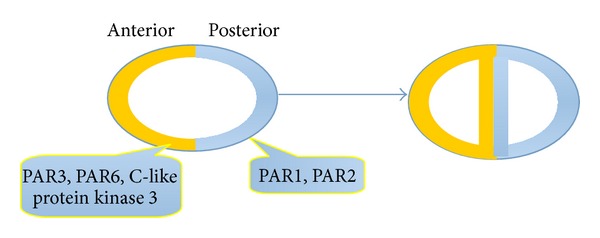
PAR protein complexes including PAR-3, PAR-6, and C-like protein kinase 3 were asymmetrically located and involve asymmetric cell division in* C. elegans*.

In* C. elegans*, PAR proteins were similarly asymmetrically located. PAR-3, PAR-6, and C-like protein kinase 3 accumulate at the anterior cell cortex of the* C. elegans* zygote when the first division occurs, whereas PAR-1 and PAR-2 accumulate posteriorly [76, 77, 79]. PAR protein complexes are also needed in* C. elegans* for other aspects of asymmetric cell division, such as the orientation and position of the mitotic spindle. The two daughter cells have different sizes and fates, but the mechanisms generating asymmetry are similar to those in neuroblasts. PAR-3, PAR-6, and PKC-3 are initially located on the cortical side and then concentrate on the anterior side after fertilization [80]. PAR-1 and PAR-2 become enriched in the posterior, noncontracting cell cortex, and inhibitory interactions between the anterior and posterior PAR proteins ensure that the groups maintain their localization to opposite cortical domains. PAR-2 prevents the cortical localization of PKC-3 [81], and PKC-3 phosphorylates PAR-2. PAR proteins in* C. elegans* are involved in regulating both asymmetric cell division and the symmetry-breaking events that establish the anteroposterior axis in the zygote [81], which is different from their functions in* D. melanogaster *(Figure 4).

## 9. Future Perspectives

Eukaryotes have a well-regulated and orderly growth with a low frequency of mutation via mitosis [82]. Conversely, in prokaryotes and unicellular eukaryotes, cells divide by amitotic processes, including budding. Although mitosis prevails in complex eukaryotes, endocycle involving multiple rounds of DNA replication without intervening mitosis step is an evolutionarily conserved means of generating multinucleated cells [33, 36, 37]. The process of PGCCs generating daughter cells through budding is very different from the traditional mitotic growth of eukaryotic diploid cells [5], which is regulated by many kinds of cell-cycle related proteins and Cdc42 [3]. PGCCs thus use budding from simple organisms and may demonstrate the ability to use an evolutionarily conserved process for renewal and fast reproduction.

In recent years, many of the key questions in asymmetric cell division have been answered. Despite these major advances, we still lack a molecular understanding of many of the processes involved. Furthermore, we still have no real clue as to how asymmetric cell division is regulated in mammalian adult stem cell lineages. Researchers have found that there is a link between the dysregulated asymmetric cell division of stem cells and tumorigenesis in mammals. Neuroblasts fail to differentiate in* D. melanogaster* embryos, leading to tumor-like overproliferation. After they have been transplanted into the abdomen of another fly, the tumors continue to grow, metastasize, and become aneuploid. The detailed mechanism of PGCCs generating daughter cells via budding is still unclear. Budding in yeast,* D. melanogaster*, and* C. elegans *may be served as the model to understand the potential mechanism of asymmetric division in PGCCs. In future, more studies of understanding the contribution of asymmetric cell division of PGCCs to mammalian development and tumorigenesis will be the primary goal.
